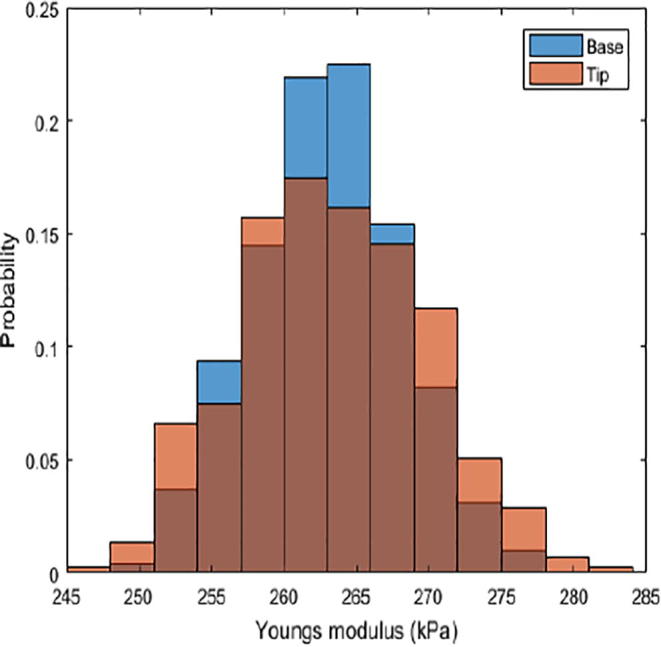# Patterning of human epidermal stem cells on undulating elastomer substrates reflects differences in cell stiffness

**DOI:** 10.1016/j.actbio.2019.01.063

**Published:** 2019-03-15

**Authors:** Seyedeh Atefeh Mobasseri, Sebastiaan Zijl, Vasiliki Salameti, Gernot Walko, Andrew Stannard, Sergi Garcia-Manyes, Fiona M. Watt

**Affiliations:** a Centre for Stem Cells and Regenerative Medicine, King's College London, 28th Floor, Tower Wing, Guy's Hospital, Great Maze Pond, London SE1 9RT, United Kingdom; b Department of Biology and Biochemistry, University of Bath, Claverton Down, Bath BA2 7AY, United Kingdom; c Department of Physics and Randall Institute of Cell and Molecular Biophysics, King’s College London, WC2R 2LS, United Kingdom

**Keywords:** Epidermal stem cells, Topographical substrates, AFM, Cell-cell junctions

## Abstract

In human skin the junction between epidermis and dermis undulates, the width and depth of the undulations varying with age and disease. When primary human epidermal keratinocytes are seeded on collagen-coated polydimethylsiloxane (PDMS) elastomer substrates that mimic the epidermal-dermal interface, the stem cells become patterned by 24 h, resembling their organisation in living skin. We found that cell density and nuclear height were higher at the base than the tips of the PDMS features. Cells on the tips not only expressed higher levels of the stem cell marker β1 integrin but also had elevated E-cadherin, Desmoglein 3 and F-actin than cells at the base. In contrast, levels of the transcriptional cofactor MAL were higher at the base. AFM measurements established that the Young’s modulus of cells on the tips was lower than on the base or cells on flat substrates. The differences in cell stiffness were dependent on Rho kinase activity and intercellular adhesion. On flat substrates the Young’s modulus of calcium-dependent intercellular junctions was higher than that of the cell body, again dependent on Rho kinase. Cell patterning was influenced by the angle of the slope on undulating substrates. Our observations are consistent with the concept that epidermal stem cell patterning is dependent on mechanical forces exerted at intercellular junctions in response to undulations in the epidermal-dermal interface.

**Statement of significance:**

In human skin the epidermal-dermal junction undulates and epidermal stem cells are patterned according to their position. We previously created collagen-coated polydimethylsiloxane (PDMS) elastomer substrates that mimic the undulations and provide sufficient topographical information for stem cells to cluster on the tips. Here we show that the stiffness of cells on the tips is lower than cells on the base. The differences in cell stiffness depend on Rho kinase activity and intercellular adhesion. We propose that epidermal stem cell patterning is determined by mechanical forces exerted at intercellular junctions in response to the slope of the undulations.

## Graphical abstract

**Figure:**
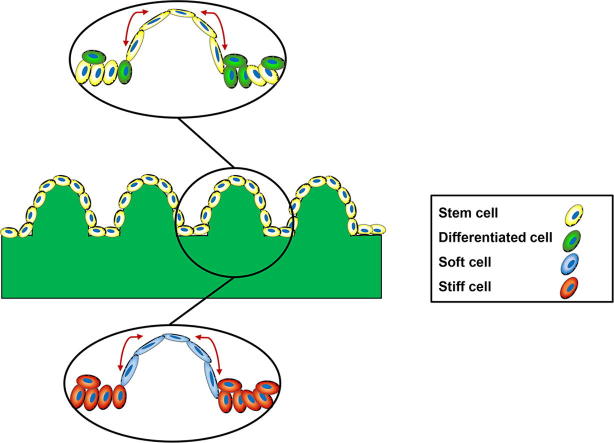

## 1 Introduction

Mammalian skin is built from two histologically and physiologically distinct tissue compartments: an epithelial layer called the epidermis and an underlying connective tissue layer called the dermis. In humans, the interface between the epidermis and dermis is not flat but undulates [1]. The interfollicular epidermis (IFE) comprises multiple cell layers, with the stem cell compartment attached to an underlying basement membrane [2] and cells undergo terminal differentiation as they move through the suprabasal layers [3]. Extrinsic signals such as interactions with neighboring cells, extracellular matrix (ECM) adhesion, tissue stiffness and secreted factors are known to regulate the behavior of stem cells [2]. Physical forces such as cell shape, shear forces and substrate stiffness all affect the balance between stem cell proliferation and differentiation [4]. Internal and external mechanical loading affects the biology of both epidermis and dermis and is mediated through mechanochemical transduction processes that involve both cell-cell and cell-ECM adhesion [5].

The importance of physical parameters has been explored by seeding individual epidermal cells (keratinocytes) on ECM-coated micro-patterned islands. Restricting keratinocyte spreading on 20 μm diameter circular islands triggers terminal differentiation whereas cells on 50 μm diameter islands remain spread and do not differentiate [6], [7]. On larger islands, that can accommodate approximately 10 cells, keratinocytes form a stratified ‘micro-epidermis’ with stem cells in the basal layer and differentiated cells (which express markers such as involucrin and transglutaminase 1) in the suprabasal layer. Actin polymerisation, desmosomes and adherens junctions are key mediators of micro-epidermis assembly [7].

Several of the signal transduction pathways that regulate keratinocyte differentiation in response to physical cues have been identified [8]. One of the key mechanotransduction mechanisms is YAP/TAZ signalling. The subcellular localisation of YAP and TAZ is controlled by surface topography, ECM stiffness and cell shape. YAP and TAZ translocate between nucleus and cytoplasm in response to mechanical cues [9]. Another key pathway is mediated by the SRF (serum-response factor) transcription factor, which is regulated by RhoA, actin polymerisation and the transcriptional cofactor MRTF-A (MAL). Actin polymerisation controls translocation of MAL into the nucleus in response to cell-ECM and cell-cell adhesion [10]. MAL and SRF mediate shape induced terminal differentiation of individual keratinocytes [11], while YAP/TAZ signalling in keratinocytes is regulated by intercellular adhesion [12].

In human epidermis the cells in the basal layer are patterned, with stem cells expressing highest levels of β1 integrin clustered where the basal layer comes closest to the skin surface. We are able to mimic the undulations by creating collagen-coated PDMS substrates and have shown that the topography that most closely resembles healthy human skin induces stem cell clustering, with nuclear YAP, on the tips [13], [12]. Patterning of stem cells and nuclear YAP can be disrupted by treating cells with a Rho kinase inhibitor (Y-27632), a Non-muscle Myosin 2 inhibitor (Blebbistatin) or by preventing the assembly of desmosomes and adherens junctions by reducing the extracellular calcium ion concentration [12].

Although ECM adhesion is known to regulate both the spatial location and the differentiation of keratinocytes in vivo and in vitro [1], [2], it is hard to envisage how a uniform collagen coating could determine the patterning of stem cells on undulating topographies. An alternative explanation, and one that is attractive given the response of the YAP pathway to the topographies [12], would be that patterning is controlled by the forces exerted by neighbouring cells, which could, in turn, depend on the slope of the undulations. In the present study, we therefore used the undulating collagen-coated PDMS substrates to explore the potential physical and cell biological mechanisms by which stem cells cluster on the tips of features. Our study reveals marked differences in cell stiffness in different locations and suggests that forces mediated by cell-cell adhesion in response to the slopes of the undulations determine stem cell patterning.

## 2 Materials and methods

### 2.1 Substrate preparation

Patterned and flat polydimethylsiloxane (PDMS) substrates were prepared as previously described [13], [14]. Briefly, the molding precursor, made of polymerised hydroxyethyl methacrylate (PHEMA), was cast on a methacrylated glass slide. By using a photomask and UV light, the PHEMA films were crosslinked and the negative pattern was created. The PDMS precursor was then mixed with the curing agent (10:1 ratio by weight, Sylgard 184s Silicone Elastomer Kit, Dow Corning) and cast on the mold. The PDMS was cured in an oven at 80 °C and then peeled off from the mold. Prior to cell culture, the patterned PDMS substrates were sterilised using 70% ethanol and washed with PBS, followed by collagen coating (100 μg mL^−1^ rat tail type 1 collagen, from Becton Dickinson) by adsorption for 2 h at 37 °C.

### 2.2 Keratinocyte culture

Primary human keratinocytes (strain km) were cultured on mitotically inactivated J2 3T3 cells in complete FAD medium (consisting of one part Ham’s F12 medium, three parts Dulbecco’s modified Eagle’s medium (DMEM), 1.8 × 10^−4^ M adenine, 10% fetal calf serum (FCS), 0.5 μg mL^−1^ hydrocortisone, 5 μg mL^−1^ insulin, 10^−10^ M cholera toxin and 10 ng mL^−1^ epidermal growth factor). Low calcium complete FAD contained calcium-free F12 and DMEM supplemented with Chelex 100 (BioRad)-treated FCS [15].

Collagen-coated patterned and flat PDMS substrates were used to culture keratinocytes at a seeding density of 150,000 per cm^2^. After 45 min at 37 °C non-adherent cells were removed and the substrates were transferred to 12-well plates containing J2 3T3 feeders at a density of 20,000 cells per cm^2^. In some experiments cells were treated with Rho kinase (ROCK) pathway inhibitor Y-27632 (Life Science Enzo) at a final concentration of 10 μM.

### 2.3 Scanning electron microscopy (SEM)

PDMS substrates seeded with keratinocytes were fixed with 2.5% glutaraldehyde in 0.1 M phosphate buffer for 2 h at room temperature. Samples were washed for 2 × 10 min in phosphate buffer. The samples were dehydrated with a gradient of 30%, 50%, 70%, 90% and 3 × 100% ethanol for 15 min at each step. Samples were then dried using hexamethyldisilazane:ethanol (1:1) for 5 min, mounted on an aluminum stub and sputter gold coated (4 nm thickness). Image acquisition was performed using a desktop SEM (JEOL NeoScope JCM 6000Plus).

### 2.4 Immunofluorescence microscopy

Cells seeded on PDMS substrates were fixed in 4%PFA for 15 min followed by three washes in PBS. Permeabilisation and blocking (PB) buffer (0.5% skimmed milk powder, 0.25% fish skin gelatin, 0.5% TritonX100, 1× HEPES-buffered saline) was added and incubated at room temperature for 1 h. Substrates were incubated in primary antibodies for 1 h at room temperature. Samples were washed three times in PBS and incubated with secondary antibodies at room temperature for 1 h. Samples were washed three times in PBS and then mounted on glass slides in Prolong antifade mounting reagent (Life technologies) containing DAPI. Samples were imaged using a Nikon A1 upright confocal microscope with a 20× objective. Images are presented as maximum intensity projections.

Primary antibodies used were as follows: anti-β1 integrin mouse monoclonal (clone P5D2; 1:500), anti-involucrin rabbit polyclonal (DH1; 1:200), anti-E-Cadherin mouse monoclonal (clone HECD1; 1:200), anti-Desmoglein 3 mouse monoclonal (Abcam; 1:25), anti-MAL rabbit polyclonal (Abcam; 1:200), anti-Ki67 rabbit polyclonal (Abcam; 1:200), and anti-YAP mouse monoclonal (Santa Cruz; 1:500). Alexa Fluor™ 488 Phalloidin (ThermoFisher Scientific; 1:1000) was also used. Secondary antibodies of the appropriate species were conjugated to Alexa Fluor™ 488, 555 and 647 (ThermoFisher Scientific).

### 2.5 Atomic force microscopy (AFM)

AFM synchronised with optical microscopy (BioScope Resolve™ BioAFM, Bruker) was used to assess the biomechanical properties of the cells. Keratinocytes were seeded on substrates 24 h prior to analysis and measurements were conducted at 37 °C in complete FAD medium. Optical microscopy was used to localise the AFM tip on the top or base of PDMS topographical features. Silicon nitride MLCT cantilevers (Bruker) with nominal spring constants of 0.02 N/m (MLCT-B, for undulating substrates) and 0.6 N/m (MLCT-F, for flat substrates) were used. Both cantilever types have pyramidal tips with effective semi-included angles of 18° and a nominal radius of curvature of 20 nm. The size for each force-volume scan was selected in the range of 15–50 μm^2^ with 256 (=16 × 16) or 1024 (=32 × 32) force-separation measurement acquired. Each force-separation measurement was acquired with a maximum ramp size of 6 µm and ramp rate 0.5 Hz, up to a maximum force of 2 nN. Analysis of force-volume scans to obtain the Young’s modulus maps was performed with Nanoscope Analysis software (Bruker) using the linearized Sneddon conical model over the force range 0.4–1.6 nN and assuming a Poisson’s ratio of 0.5.

### 2.6 Live cell imaging

Keratinocytes were seeded on collagen-coated PDMS substrates 24 h prior to live imaging. In some experiments, keratinocytes were infected with lentiviral vectors encoding GFP (pLenti-CMV GFP puro (658-5), gift from Eric Campeau, Addgene plasmid # 17448,) and mCherry (pLV-mCherry, gift from Pantelis Tsoulfas, Addgene plasmid # 36084). Keratinocytes were transduced with lentiviral plasmids carrying GFP and mCherry reporters 2 days prior to live imaging. A JuLI™ Stage Real-Time Cell History Recorder device was used to acquire images from live keratinocytes. Images were captured with an interval time of 1–2 h and videos were created using JuLI™ Stage edit software.

### 2.7 Statistical analysis and image quantification

Prism7 GraphPad software was used to generate graphs and perform statistical analysis. All data were analysed by unpaired or paired ANOVA or paired *t*-test and presented as the mean ± standard deviation. The number of replicate experiments is reported in the figure legends.

The immunofluorescence labeled images were processed by imageJ and presented as maximum intensity projections. Using imageJ, cells labelled with DAPI, Ki67, involucrin or other markers were quantified and presented as a percentage of positive cells. Nuclear height and volume were measured using the 3D object counter plugin.

## 3 Results

### 3.1 Seeding keratinocytes on undulating topographies

**Fig. 1:**
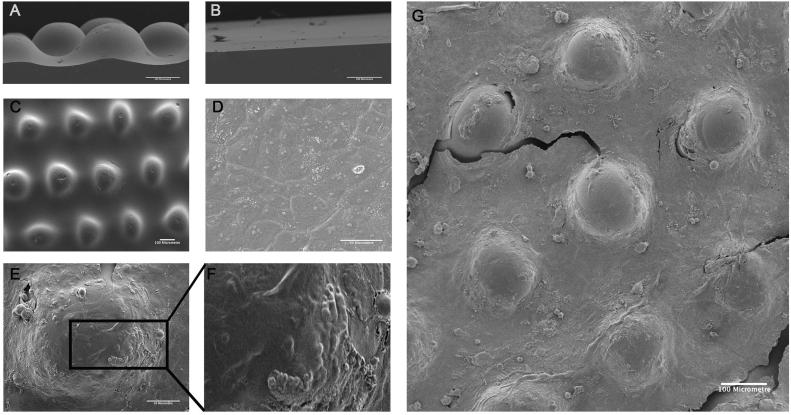
SEM of keratinocytes cultured on PDMS substrates. (A, B) Side views of (A) patterned and (B) flat PDMS substrates prior to collagen coating. (C) top view of patterned PDMS substrate prior to collagen coating. (D-G) Keratinocytes on (D) flat and (E, F, G) undulating PDMS substrates. (F) is higher magnification view of boxed region in (E). Scale bars: 100 μm (A, B, C, G), 50 μm (D, E).

We previously generated a set of 9 PDMS substrates (including one flat control substrate) by using three different photomasks and three different UV exposure times [13]. Differences in topography wavelength were determined by the photomask used, while the amplitude was controlled by UV exposure. The topography that best mimicked the undulations of healthy human skin (S1 in [13]) had centre-to-centre (l) and circle diameter (d) equal to 300 and 150 μm (Fig. 1A and C). Although PDMS is hydrophobic, we have previously shown that it can be coated with collagen by adsorption [13]. This topography and a flat PDMS control (S6 in [13]) (Fig. 1B) were coated with collagen and keratinocytes were allowed to attach for 45 min, ensuring enrichment for stem cells. After 24 h SEM revealed that both substrates were covered by a confluent multilayered sheet of keratinocytes (Fig. 1D and E). However, whereas cell morphology was rather uniform on the flat substrate (Fig. 1D), cell morphology was more heterogeneous on the undulating substrate (Fig. 1E–G).

### 3.2 Topographical features dictate the location of stem cells and differentiated cells, and influence cell movements

**Fig. 2:**
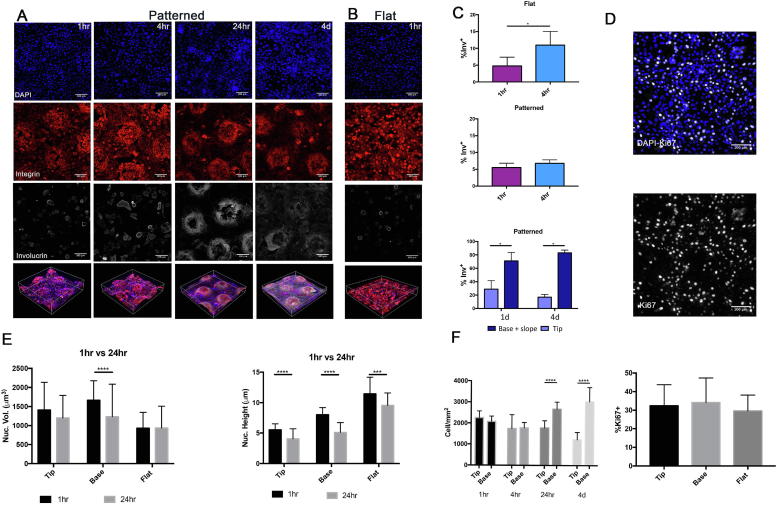
Effect of undulating topography on keratinocyte proliferation, differentiation and morphology. (A, B, D) Representative images (maximum intensity projections) of cultured keratinocytes labelled for the markers indicated on (A, D) patterned and (B) flat PDMS for the length of time shown. Scale bars: 100 μm. (C, F) Quantification of percentage of involucrin positive cells (% Inv^+^) (C), Ki67+ cells (F) cell density (F). The total % involucrin-positive cells per topography regardless of location is shown in the middle panel of (C). (E) Quantitation of nucleus volume (nucl. Vol.). *P < 0.05, *** P < 0.001, ****P < 0.0001. (n = 3, 9 images (each corresponding in area to 0.40 mm^2^) per group were analysed).

To examine the evolution of cell patterning on the undulating substrates, keratinocytes were cultured on the collagen-coated patterned and flat PDMS substrates for 1 h, 4 h, 24 h and 4 days (Fig. 2). β1 integrin bright cells clustered on tips of the undulating topography as early as 1 h and the pattern was pronounced from 4 h to 4 days (Fig. 2A; Suppl. Fig. 1). The percentage of involucrin-positive differentiated cells was 5.5% and 6.7% at 1 h and 4 h time points, respectively (Fig. 2C), confirming that the majority of adherent cells were undifferentiated. The number of differentiated cells was significantly higher at the base and slopes of the features than the tips at both time points (Fig. 2C), consistent with earlier observations [13]. When we compared the base and slopes separately there were more involucrin-positive cells on the slopes than the base at 24 h (Suppl. Fig. 1B).

Keratinocyte motility has been reported to distinguish stem cells from differentiated cells [16], with stem cells having the highest rotational speed. It has also been suggested that rotational motion of individual keratinocytes results in collective motion of stem cells [17]. To determine whether on undulating substrates cells differentiated according to their location or whether there was significant cell movement, we collected time-lapse images. We mixed cells that had been labelled with GFP or mCherry lentiviral vectors to facilitate observation of cell-cell interactions. The cultures also contained unlabeled cells (Movie 1).

Initially cells were observed moving on the base and tips of the substrates; however, when cells reached confluence movement largely ceased. Prior to confluence GFP and mCherry labeled keratinocytes were observed not only moving relative to one another but also sliding over one another, indicating that once cells have differentiated and left the basal layer they can translocate from their original position (Movie 1). By lower power time-lapse imaging of unlabeled cells, we could detect more movement on the bases than the tips, with movement resembling a push and pulling pattern (Movie 2). No apoptotic cells were observed by time-lapse imaging.

These results suggest that patterning is achieved by cues provided by the topography of the culture substrate rather than by cell sorting due to cell migration.

### 3.3 Topographical features dictate cell density and nuclear morphology

Mechanical forces experienced by cells can lead to nuclear deformation and flattening, and mechanosensing pathways are regulated by active and passive transport of proteins through nuclear pores [18]. To determine whether, like cell shape (Fig. 1), nuclear morphology was affected by substrate topography, we measured nuclear height and volume in cells at the tips and base 1 h and 24 h after seeding (Fig. 2E). We compared cells on patterned and flat topographies. Overall, there was no difference in nuclear volume at either time point and in any location, the exception being a reduction in volume of cell nuclei at the base of features between 1 h and 24 h. Nuclear height was reduced after 24 h, regardless of cell location. In addition nucleus height reflected cell position, being highest in cells on flat surfaces, and lowest in cells on the tips of undulating substrates (Fig. 2E). The nuclear volume of attached cells on the tips and base was comparable (Fig. 2E).

At 1 h and 4 h there was no difference in cell density between tips and bases, but from 24 h onwards cells were significantly more crowded on the bases (Fig. 2F). Quantification of cell proliferation as the percentage of Ki67 positive cells confirmed that, as reported previously [12], there were no differences in proliferation between cells at tips and bases (Fig. 2D). The differences in cell density and nuclear morphology are thus more likely to reflect differences in cell shape (Fig. 1).

### 3.4 Influence of surface topography on the actin cytoskeleton, intercellular adhesion and MAL

**Fig. 3:**
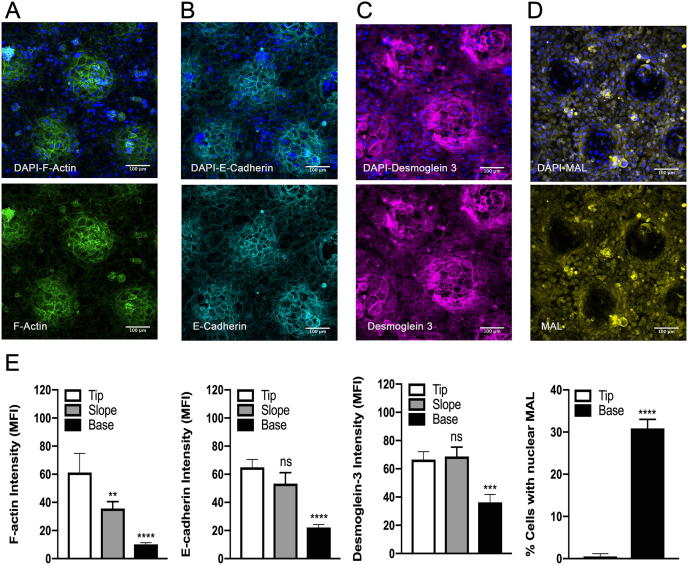
Immunofluorescence labelling of keratinocytes on patterned substrates 24 h post seeding. Representative images (maximum intensity projections) showing expression of F-actin (A), E-cadherin (B), Desmoglein 3 (C) and MAL (D) with DAPI counterstain (blue). Scale bars: 100 μm. (E) Quantitation of immunofluorescence images. **P < 0.01, ***P < 0.001, ****P < 0.0001. n = 3 independent experiments, 9 images (each corresponding in area to 0.40 mm^2^) per experiment. one-way ANOVA for E-cadherin, F-actin, Desmoglein 3 and paired *t*-test for MAL.

The differences in the shape and motility of cells on tips and base of the topographies suggested there would be corresponding differences in the cytoskeleton and intercellular junctions. At 24 h we observed higher F-actin levels in cells on the tips than on the bases, correlating with the location of β1 integrin bright cells (Fig. 3A and E). E-cadherin, a marker of adherens junctions, and Desmoglein 3, a desmosome marker, were also expressed at higher levels tips (Fig. 3B, C, and E). In contrast, keratinocytes attached on the tips expressed lower levels of MAL compared to cells at the base (Fig. 3D and E).

### 3.5 Influence of surface topography on cell stiffness

**Fig. 4:**
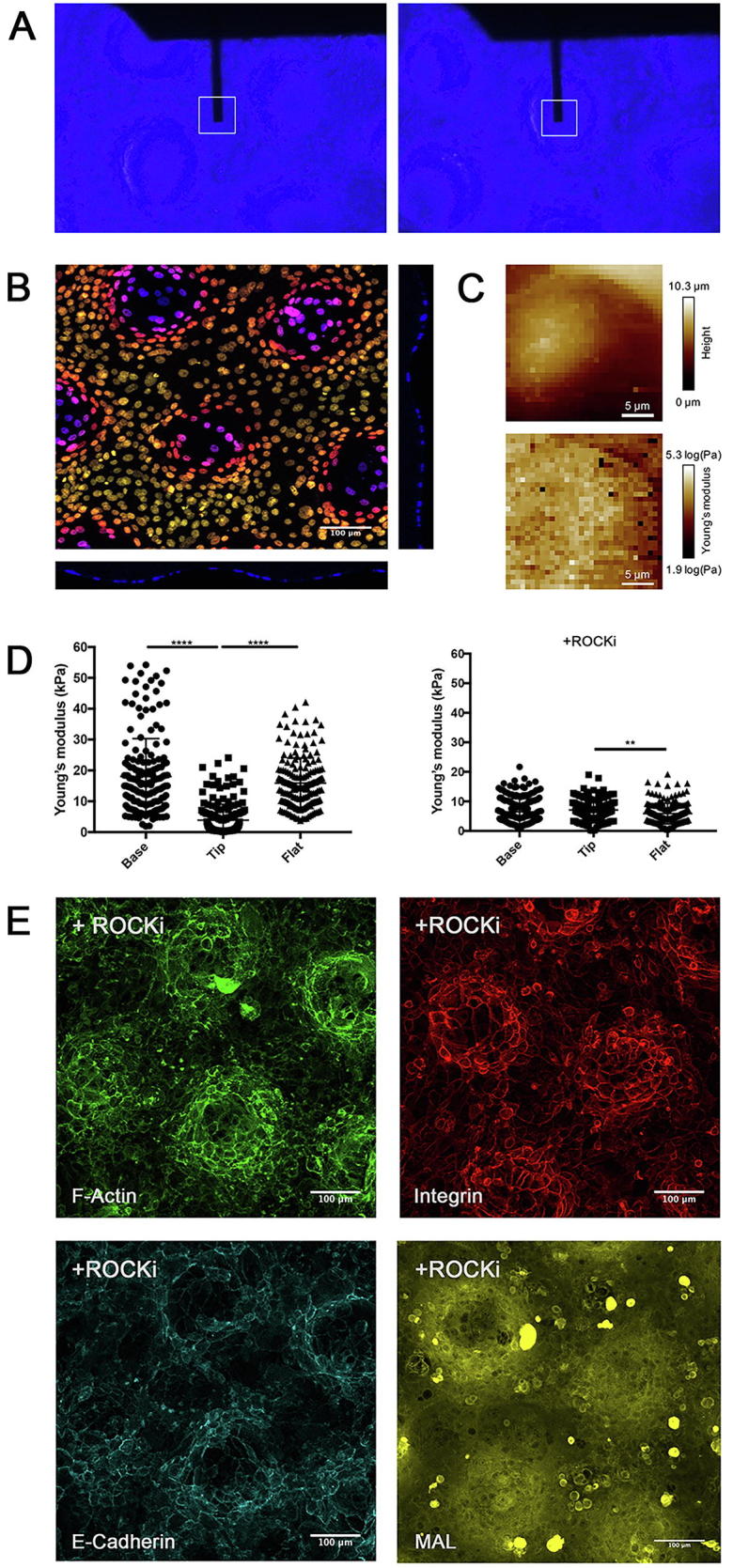
Biomechanical properties of keratinocytes and the effect of Rho kinase inhibition. (A) AFM measurements of keratinocytes were carried out at different positions on undulating substrates by positioning the cantilever tip at the tips or base of substrates using an optical microscope. (B) Representative image of depth-coding of Z stack and cross section of DAPI channel showing cultured keratinocytes on patterned PDMS substrate. (C) Representative images of height and Young’s modulus maps. Each square corresponds to one force displacement curve. (D) Quantification of Young’s modulus measurements of keratinocytes on patterned (tip, base) and flat PDMS substrates 24 h post seeding in the presence or absence of ROCKi. ** P < 0.01, *** P < 0.001. (n = 3, 9 random (15 × 15 μm) areas per group, 20 measurements per area) (E) Representative images (maximum intensity projections) of keratinocytes 24 h post seeding in the presence of ROCKi labelled for the markers shown. Scale bars: 100 μm (n = 3, 9 images per group were analysed).

Since physical forces are transmitted between epithelial cells via intercellular junctions [5] and keratins provide mechanical strength against cellular deformation [19], we hypothesised that cell stiffness might vary according to cell position on the patterned topographies. We therefore used AFM to measure the Young’s modulus of keratinocytes in different regions of the substrate (Fig. 4A–C). The nature of the force-volume AFM data precluded obtaining stiffness measurements with subcellular resolution (Fig. 4A) and therefore the measurements represent the average for the entire cell (nucleus, cytoplasm and intercellular junctions) in each location on the topography. We could only make measurements of cells on the tips and base, not on the slopes. We did not find any difference in the stiffness of PDMS in different regions of the topography (Suppl. Fig. 2).

Quantification of Young’s modulus revealed that the stiffness of cells on the tips (3.9 ± 5.2 kPa) was significantly lower than that of cells on the base (18.0 ± 12.3 kPa) (Fig. 4D). The stiffness of cells on the flat substrates (15.9 ± 8.2 kPa) was comparable to cells on the base of patterned substrates. Treatment with a Rho kinase inhibitor (ROCKi) reduced the Young’s modulus of cells in all experimental groups. Furthermore, in the presence of the inhibitor, the Young’s modulus of cells on tips (7.1 ± 3.4 kPa) and base (6.5 ± 4.4 KPa) was comparable (Fig. 4D).

Cell shape and ROCK regulate differentiation of epidermal stem cells [6]. Inhibition of Rho GTPase signaling disrupts adherens junction and stimulates keratinocyte colony expansion in culture [12] (Fig. 4E). In the presence of ROCKi, clusters of integrin bright cells remained at the tips, which expressed high levels of F-Actin and low levels of MAL. However, E-cadherin staining was disrupted, confirming the requirement for Rho kinase activity to maintain adherens junctions.

### 3.6 Differential stiffness of cell-cell junctions and the cell body

**Fig. 5:**
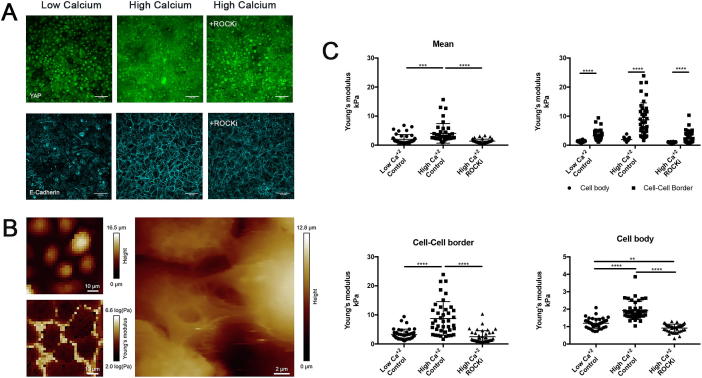
Biomechanical properties of the cell body and cell-cell junctions of keratinocytes cultured on flat substrates. (A) Representative images of immunolabled keratinocytes following culture in low or high calcium FAD, or high calcium FAD containing ROCKi. Scale bars: 100 μm. (B) Representative images of height and Young’s modulus map. (C) Quantitation of mean Young’s modulus of cell-cell borders and cell body. **P < 0.01, ***P < 0.001, ****P < 0.0001. (n = 3, 9 random (50 × 50 μm) areas per group, 20 measurements per area).

To examine the influence of cell-cell junctions on biomechanical properties, keratinocytes were cultured on collagen-coated glass in low calcium FAD medium for 48 h to impair the formation of E-cadherin mediated adherens junctions. Following AFM measurements, the medium was changed standard (high calcium) FAD. In a separate set of samples, ROCKi was added to standard FAD. Cells were incubated overnight and the AFM measurements were repeated. High levels of nuclear YAP and low levels of membrane E-cadherin expression were observed in low calcium FAD. After increasing the calcium concentration, YAP expression localised in the cytoplasm and E-cadherin expression increased significantly (Fig. 5A).

The AFM measurements indicated that the cell body of keratinocytes cultured in low calcium FAD displayed a lower Young’s modulus (2.0 ± 1.6 kPa) than keratinocytes cultured in standard medium. Switching cells from low calcium to standard medium led to a significant increase in cell body stiffness (4.1 ± 3.4 kPa), which could be prevented by ROCKi (1.4 ± 0.7 kPa) (Fig. 5C). Under all experimental conditions, the stiffness of the cell body was significantly lower than that of cell-cell borders (Fig. 5C).

### 3.7 Effect of surface topography on cell-cell junctions

**Fig. 6:**
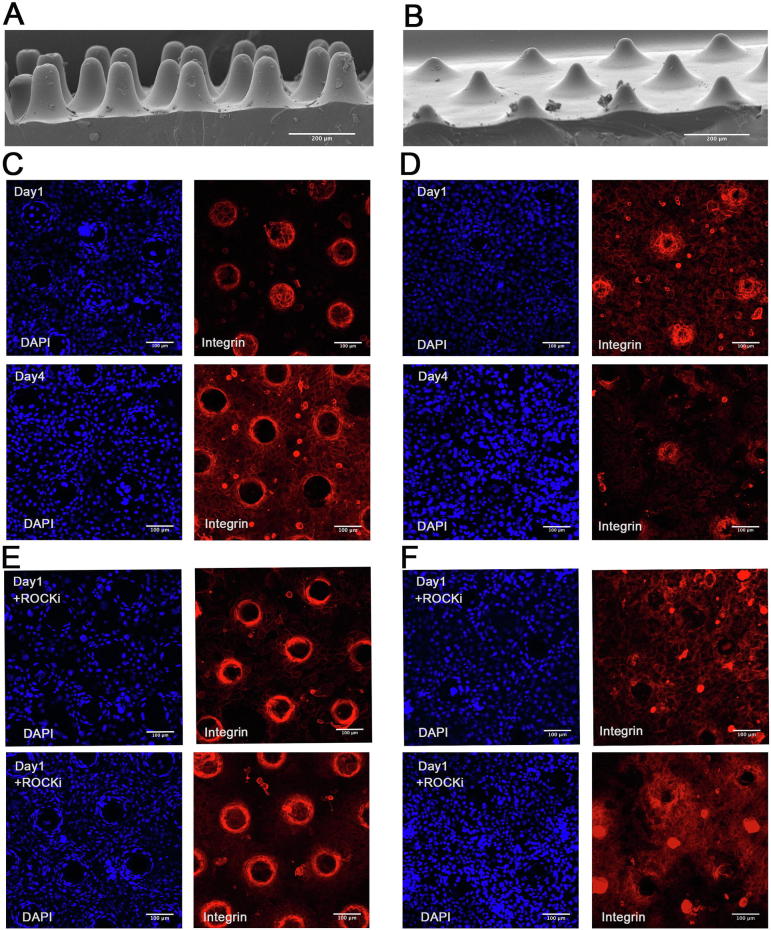
Effect of culturing keratinocytes on topographies with different slopes. (A) SEM images of uncoated patterned PDMS with steep (A) and shallower (B) slopes. (C-F) Representative images (maximum intensity projections) of keratinocytes immunolabelled for β1 integrin (red) with DAPI counterstain (blue). Cells were cultured for the number of days shown in the presence or absence of ROCKi. (C, E) steep, (D, F) shallow slopped topographies. Scale bars: 200 μm (A, B), 100 μm (C–F). (n = 3, 9 images per group (each corresponding in area to 0.40 mm^2^) were analysed).

We hypothesised that stem cell patterning is controlled by forces exerted by cells on the slopes of the topographical features, rather than the tips or bases. To evaluate this, two additional topographies [12] were used. Keratinocytes seeded on a substrate with steep features (Fig. 6A; S5 in [12]) formed an intact sheet at day 1, but the cells subsequently retracted from the tips and by day 4 there were no longer any cells on the tips (Fig. 6, C). This effect – i.e. extent of retraction - was less substantial for a substrate with shallower slopes (Fig. 6B, D; S9 in [12]). By disrupting cell-cell junctions with ROCKi, keratinocytes failed to form a confluent sheet covering the tips, even on the substrate with the shallower slope (Fig. 6E and F).

## 4 Discussion

When primary human keratinocytes are seeded on a collagen-coated undulating polydimethylsiloxane (PDMS) elastomer substrate that mimics the topography of the epidermal-dermal junction in healthy, young skin, the cells become patterned such that the β1 integrin bright stem cells localise to the tips of the features [13]. Here we show that the cells at the tips of the features are softer than those at the base, correlating with enhanced accumulation of E-cadherin, Desmoglein 3 and F-actin at cell-cell borders. The differential stiffness of the cells was dependent of Rho kinase activity. Our observations lead us to propose that epidermal stem cell patterning is achieved through forces exerted on stem cells by cells on the slopes, mediated by intercellular adhesion. This would be consistent with earlier work showing that keratinocytes are stiffer than other cell types because of their keratin intermediate filament network [20], and that mutations or antibody treatments that impair desmosomes alter the viscoelastic properties of keratinocytes [21], [22], [23].

While the dimensions of the PDMS substrates precluded comparing forces at intercellular junctions, we were able to measure them on flat substrates. We observed that intercellular junctions were stiffer than cell bodies and that their stiffness depended on Rho kinase. In future it would be interesting to discover whether forces exerted via intercellular junctions differ according to the slope of the undulations.

We have previously reported that tip cells have nuclear YAP, a localisation that is dependent on Rho kinase and intercellular adhesion [12]. We now report that cells at the base of the topographies have higher levels of MAL, again dependent on Rho kinase. Given that YAP and MAL/SRF differentially regulate differentiation [12], [6], the localisation of MAL is consistent with the higher frequency of differentiation at the base of the substrates. Nevertheless, the fact that basal and suprabasal keratinocytes were able to move relative to one another indicates that the accumulation of involucrin-positive cells at the base of the features [13] could be due to a combination of MAL/SRF signalling in the basal layer and movement of suprabasal cells relative to the underlying basal cells.

We have recently created a dynamic rig and shown that topographical features can impose patterning on a flat sheet of cells within 48 h [24]. On the dynamic substrates stem cell clustering can be induced independent of the location of differentiating, involucrin-positive cells. This is consistent with the finding that differentiating cells can move relative to underlying basal cells, both in culture and during wound healing [25], [26]. In addition, studies of keratinocytes growing on the dynamic rigs lead us to speculate that it is the undulations rather than whether cells are on tips or base that is important in determining stem cell patterning. We provide further indirect support for this with the finding that the ability of integrin-bright stem cells to localise to the tips of PDMS substrates depends on the angle of the slope.

Crowding in the epidermal basal layer, which we observed on the base of the topographies, is known to affect cell shape and can trigger movement into the suprabasal layer through a decrease in cortical tension and increased cell-cell adhesion [27], [28]. We speculate that the crowding of cells on the base of the topographies is due to cells being pulled from the tips. It is possible that the differences in cell stiffness between tips and base reflect differences in cell crowding. It is important to emphasise that in our experimental model it is the stiffness of the cells not the substrate that is patterned.

Our finding that topography affects cell stiffness has implications for the changes in epidermal-dermal topography that occur in human skin with age and inflammation. The undulations decrease with age [29] and increase in psoriatic lesions [30], [31]. While previous studies have focused on changes in the height of the undulations, our studies suggest that changes in the slope may also impact on the disruption of epidermal homeostasis that occurs in psoriatic lesions. In the case of corneal injury biomechanical changes in stromal cells are linked to accumulation of inflammatory cells [32] and it is therefore tempting to speculate that differences in the stiffness of epidermal cells and their junctions could lead to an altered immune infiltrate. It is also possible that materials that mimic the undulating topography of the epidermal-dermal junction, for example by casting topographies out of collagen gels, could enhance wound healing or reduce scarring.

## Figures and Tables

**Supplementary figure 1:**