# Supplementary figures and images for: Patterning of human epidermal stem cells on undulating elastomer substrates reflects differences in cell stiffness
Source: Acta Biomater. 2019 Mar 15;87:256–64. doi: 10.1016/j.actbio.2019.01.063 (PMC6401207; doi:10.1016/j.actbio.2019.01.063)

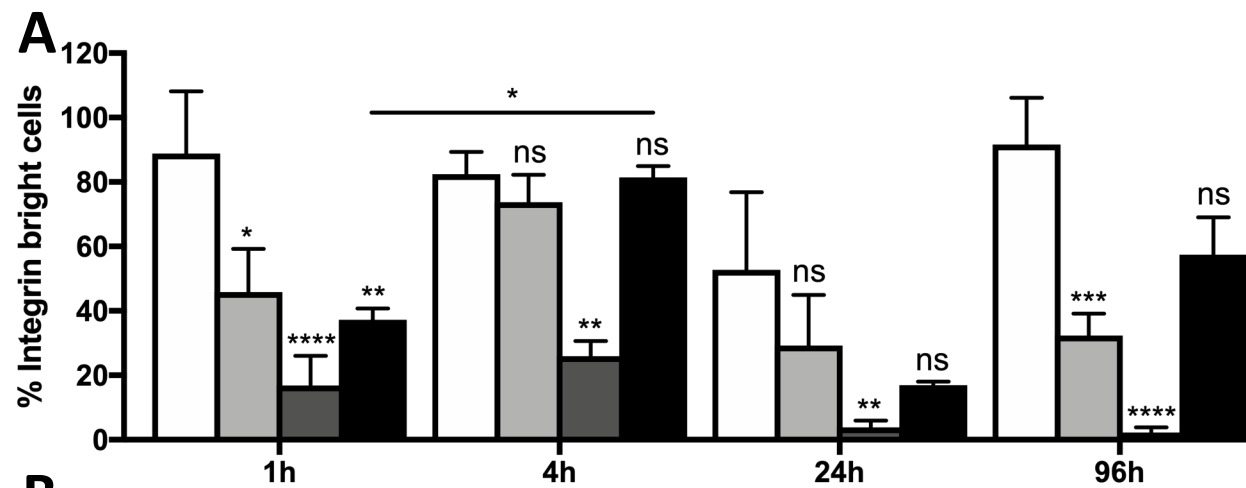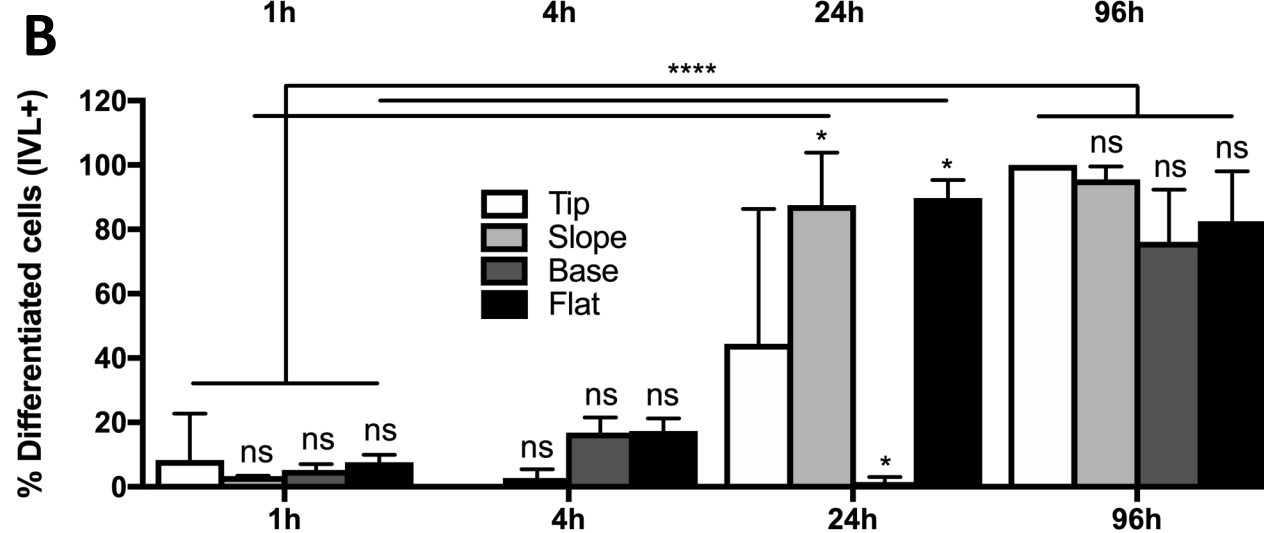

Supplement: Supplementary data 1 [file mmc1.pdf]
